# SEIR model for COVID-19 dynamics incorporating the environment and social distancing

**DOI:** 10.1186/s13104-020-05192-1

**Published:** 2020-07-23

**Authors:** Samuel Mwalili, Mark Kimathi, Viona Ojiambo, Duncan Gathungu, Rachel Mbogo

**Affiliations:** 1 School of Mathematical Sciences, Jomo Kenyatta University of Agriculture and Technology, Nairobi, Kenya; 2 Mathematics & Statistics Department, Machakos University, Machakos, Kenya; 3 Institute of Mathematical Sciences, Strathmore University, Nairobi, Kenya

**Keywords:** SEIR model, COVID-19 dynamics, Social distancing, Mathematical model, Basic reproduction number, Runge–Kutta method

## Abstract

**Objective:**

Coronavirus disease 2019 (COVID-19) is a pandemic respiratory illness spreading from person-to-person caused by a novel coronavirus and poses a serious public health risk. The goal of this study was to apply a modified susceptible-exposed-infectious-recovered (SEIR) compartmental mathematical model for prediction of COVID-19 epidemic dynamics incorporating pathogen in the environment and interventions. The next generation matrix approach was used to determine the basic reproduction number $R_0$. The model equations are solved numerically using fourth and fifth order Runge–Kutta methods.

**Results:**

We found an $R_0$ of 2.03, implying that the pandemic will persist in the human population in the absence of strong control measures. Results after simulating various scenarios indicate that disregarding social distancing and hygiene measures can have devastating effects on the human population. The model shows that quarantine of contacts and isolation of cases can help halt the spread on novel coronavirus.

## Introduction

Coronaviruses are a large family of viruses that are known to cause illness ranging from the common cold to more severe diseases such as severe acute respiratory syndrome (SARS). The severe acute respiratory syndrome coronavirus 2 (SARS-CoV-2) was identified as the cause of a cluster of pneumonia cases in Wuhan [1], a city in the Hubei Province of China, at the end of 2019. It subsequently spread throughout China and elsewhere, becoming a global health emergency. In February 2020, the World Health Organization (WHO) designated the disease coronavirus disease 2019 (COVID-19) a global pandemic [2].

The objective of this study was to develop a modified SEIR compartmental mathematical model for prediction of COVID-19 epidemic dynamics considering different intervention scenarios which might give insights on the best interventions to reduce the epidemic risk.

Several authors have worked on mathematical modeling of the novel coronavirus. A mathematical model for Middle East respiratory syndrome coronavirus (MERS-CoV) transmission dynamics was used to estimate the transmission rates in two periods due to the implementation of intensive interventions [3–6]

Further and related to this work, a Bats-Hosts–Reservoir-People transmission network model for simulating the potential transmission from the infection source to the human infection was developed [5]. This article, however, differs from [5] in the sense that (1) the compartmental models are different; (2) an additional compartment for the pathogens was included to allow for non-linear interactions between humans and the environment; and (3) thorough simulation studies were performed.

**Fig. 1:**
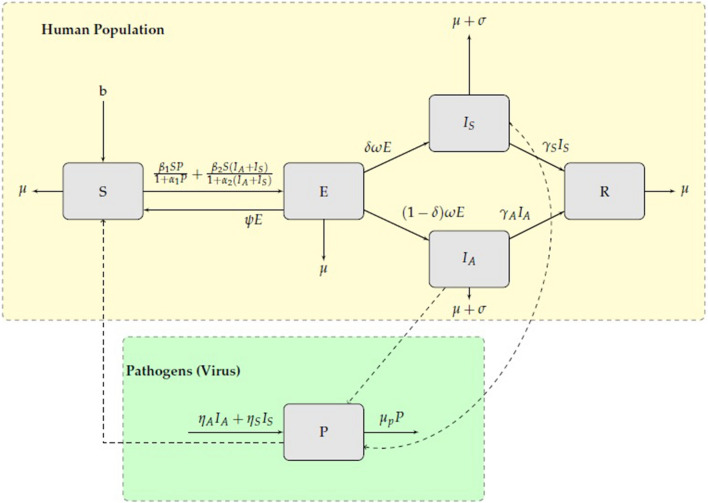
SEIR-P model of COVID-19 transmission. Depicting a human (SEIR, yellow shade) and pathogen (P, green shade) compartmental model

## Main text

### Methods

In the study, a mathematical model of the spread and transmission of SARS-CoV-2 was formulated. We consider two interacting populations, the human population as hosts and the pathogens. The model subdivides the total human population size at time *t* denoted as *N*(*t*) into susceptible *S*(*t*), exposed *E*(*t*), asymptomatic infectious $$I_A(t)$$, symptomatic infectious $$I_S(t)$$ and the recovered as *R*(*t*). The pathogen in the environment is denoted as *P*(*t*). Hence for the human population we have $$ N(t)=S(t)+E(t)+I_A(t)+I_S(t)+R(t).$$

Studies have shown that the virus can be transmitted in two ways, namely: human to human and environment to human. The epidemic data indicates that both asymptomatic $I_A(t)$ and symptomatic $I_S(t)$ infected individuals spread the COVID-19 virus to susceptible persons *S*(*t*) with whom they are in close contact. In addition, when infected individuals sneeze or cough, without taking the necessary precautions, the virus spreads to the environment they are in. Since the pathogen *P*(*t*) is known to survive in the environment for some days, susceptible individuals *S*(*t*) in close contact to this environment are likely to get exposed to these pathogens, especially in the early days of the COVID-19 outbreak before hygiene protocols are implemented. In the process of disease spread, the susceptible individual first moves to the exposed population *E*(*t*) since the host has an incubation period [5]. The exposed individual moves to either asymptomatic $I_A(t)$ or symptomatic $I_S(t)$ infectious population. *P*(*t*) is the number or quantity of pathogens present during interaction of human beings at time *t*. The majority of infectious individuals recovers and move to the recovered human population *R*(*t*).

The compartmental model depicting the interaction between the human population, and the pathogens in the environment is shown in Fig. 1.

The parameters used in the COVID-19 transmission model are given in Table 1.

**Table 1:** Description of model parameters

Model parameter name	Symbol	Value
Birth rate of the human population	*b*	0.00018 days$^{-1}$
Natural human death rate	$\mu$	$4.563\times 10^{-5}$ days$^{-1}$
Human life expectancy	$\frac{1}{\mu }$	21915 days or 60 years
Natural death rate of pathogens in the environment	$\mu _P$	0.1724 days$^{-1}$
Life expectancy of pathogens in the environment	$\frac{1}{\mu _P}$	5.8 days
Proportion of interaction with an infectious environment	$\alpha _1$	0.10
Proportion of interaction with an infectious individual	$\alpha _1$	0.10
Rate of transmission from *S* to *E* due to contact with *P*	$\beta _1$	0.00414
Rate of transmission from *S* to *E* due to contact with $I_A$ and/or $I_S$	$\beta _2$	0.0115
Proportion of symptomatic infectious people	$\delta$	0.7
Progression rate from *E* back to *S* due to robust immune system	$\psi$	0.0051
Progression rate from *E* to either $I_A$ or $I_S$	$\omega$	0.09
Death rate due to the coronavirus	$\sigma$	0.0018
Rate of recovery of the symptomatic population	$\gamma _S$	0.05 days$^{-1}$ or $\frac{1}{20 \text { days}}$
Rate of recovery of the asymptomatic human population	$\gamma _A$	0.0714 days$^{-1}$
Rate of virus spread to environment by symptomatic infectious individuals	$\eta _S$	0.1 days$^{-1}$ or $\frac{1}{10 \text { days}}$
Rate of virus spread to environment by asymptomatic infectious individuals	$\eta _A$	0.05 days$^{-1}$ or $\frac{1}{20 \text { days}}$

The model culminates to a six-dimensional system of ordinary differential equations as follows.

1 $$\begin{aligned} {\left\{ \begin{array}{ll} \frac{dS}{dt}=b-\frac{\beta _1SP}{1+\alpha _1P}-\frac{\beta _2S\left( I_A+I_S\right) }{1+\alpha _2\left( I_A+I_S\right) }+\psi E-\mu S,\\ \frac{dE}{dt}=\frac{\beta _1SP}{1+\alpha _1P}+\frac{\beta _2S\left( I_A+I_S\right) }{1+\alpha _2\left( I_A+I_S\right) }-\psi E-\mu E-\omega E,\\ \frac{dI_A}{dt}=(1-\delta )\omega E-(\mu +\sigma )I_A-\gamma _AI_A,\\ \frac{dI_S}{dt}=\delta \omega E-(\mu +\sigma )I_S-\gamma _SI_S,\\ \frac{dR}{dt}=\gamma _S I_S+\gamma _A I_A-\mu R,\\ \frac{dP}{dt}=\eta _A I_A+\eta _SI_S-\mu _pP. \end{array}\right. } \end{aligned}$$

with the initial conditions: $S(0)>0, E(0)>0, I_A>0, I_S>0, R(0)=0, P(0)>0$

The human population is born into the susceptible population at a rate *b*. The terms $\beta _1SP$ and $\beta _2S\left( I_A+I_S\right)$ describes the rate at which susceptible individuals *S*(*t*) gets infected by pathogens in the environment, *P*(*t*) and from infectious humans $I_A(t)$ and $I_S(t)$ respectively. Health experts and governments have been advising people, during this outbreak, to minimize contact with infectious individuals through social distancing. Therefore in our model we propose to have new infections occur in the form $\frac{\beta _1SP}{1+\alpha _1P}$ and $\frac{\beta _2S\left( I_A+I_S\right) }{1+\alpha _2\left( I_A+I_S\right) }$ respectively, where the interaction proportions $\alpha _1$ and $\alpha _2$ denotes reciprocal of the frequency with which susceptible individuals gets infected with COVID-19 from the environment and from infectious individuals, respectively.

#### Equilibria and basic reproduction number of the SEIR-P model

The relevant equilibrium points are obtained by solving the equations in (1) when the left hand side is equated to zero.

#### Existence of disease-free-equilibrium point (DFE)

In this case $I_A=I_S=P=0$, which implies that $E=0$ and $R=0$ too. Hence we have:

2 $$\begin{aligned} 0=b-\mu S \implies S=\frac{b}{\mu }. \end{aligned}$$

Therefore DFE is given by $\left( \frac{b}{\mu },0,0,0,0,0\right)$.

#### The basic reproduction number

The basic reproduction number, usually denoted as $R_0$ defines the average number of secondary infections caused by an individual in an entirely susceptible population. This number indicates whether the infection will spread through the population or not. The next generation matrix approach is used to obtain $R_0$. Let $x=\left( E,I_{A},I_{S},P\right) ^{T}$ then the model can be written as $\frac{dx}{dt}=F\left( x\right) -V\left( x\right)$, where

$$\begin{aligned} F(x)=\left( \begin{array}{c} \frac{\beta _{1}SP}{1+\alpha _{1}P}+\frac{\beta _{2}S\left( I_{A}+I_{S}\right) }{1+\alpha _{2}\left( I_{A}+I_{S}\right) }\\ 0\\ 0\\ \eta _{A}I_{A}+\eta _{S}I_{S} \end{array}\right)&\text{ and }&V(x)=\left( \begin{array}{c} \left( \psi +\mu +\omega \right) E\\ \left( \mu +\sigma +\gamma _{S}\right) I_{S}-\delta \omega E\\ \left( \mu +\sigma +\gamma _{A}\right) I_{A}-\left( 1-\delta \right) \omega E\\ \mu _{P}P \end{array}\right) \end{aligned}$$

Evaluating the derivatives of *F* and *V* at the disease-free equilibrium point, obtained above, yields $\mathbf{FV} ^{-1}$ as below:

$$\begin{aligned} \mathbf{FV} ^{-1}=\left( \begin{array}{cccc} \frac{\beta _{2}b\delta \omega }{\mu C_{1}C_{2}}+\frac{\beta _{2}b\left( 1-\delta \right) \omega }{\mu C_{1}C_{3}} &{} \frac{\beta _{2}b}{\mu C_{2}} &{} \frac{\beta _{2}b}{\mu C_{3}} &{} \frac{\beta _{1}b}{\mu \mu _{P}}\\ 0 &{} 0 &{} 0 &{} 0\\ 0 &{} 0 &{} 0 &{} 0\\ \frac{\eta _{S}\delta \omega }{C_{1}C_{2}}+\frac{\eta _{A}\left( 1-\delta \right) \omega }{C_{1}C_{3}} &{} \frac{\eta _{S}}{C_{2}} &{} \frac{\eta _{A}}{C_{3}} &{} 0 \end{array}\right) \end{aligned}$$

where $C_{1}=\psi +\mu +\omega$, $C_{2}=\mu +\sigma +\gamma _{S}$ and $C_{3}=\mu +\sigma +\gamma _{A}$. The reproduction number, $R_0$, is the spectral radius of the product $\mathbf{FV} ^{-1}$ which is given by;

3 $$\begin{aligned} R_0=\frac{\frac{\beta _{2}b\delta \omega }{\mu C_{1}C_{2}}+\frac{\beta _{2}b\left( 1-\delta \right) \omega }{\mu C_{1}C_{3}}+\sqrt{\left( \frac{\beta _{2}b\delta \omega }{\mu C_{1}C_{2}}+\frac{\beta _{2}b\left( 1-\delta \right) \omega }{\mu C_{1}C_{3}}\right) ^{2}+\frac{4\beta _{1}b}{\mu \mu _{P}}\left( \frac{\eta _{S}\delta \omega }{C_{1}C_{2}}+\frac{\eta _{A}\left( 1-\delta \right) \omega }{C_{1}C_{3}}\right) }}{2} \end{aligned}$$

Denoting the basic reproduction numbers for human as $R_{0}^{h}$ and for pathogens as $R_{0}^{p}$, we make the following deductions:

4 $$\begin{aligned} R_{0}^{h}= & {} \frac{\beta _{2}b}{\mu C_{1}}\left[ \frac{\delta \omega }{C_{2}}+\frac{\left( 1-\delta \right) \omega }{C_{3}}\right] \end{aligned}$$

5 $$\begin{aligned} R_{0}^{p}= & {} \frac{\beta _{1}b}{\mu \mu _{P}C_{1}}\left[ \frac{\eta _{S}\delta \omega }{C_{2}}+\frac{\eta _{A}\left( 1-\delta \right) \omega }{C_{3}}\right] \end{aligned}$$

Therefore,

6 $$\begin{aligned} R_{0}=\frac{R_{0}^{h}+\sqrt{\left( R_{0}^{h}\right) ^{2}+4R_{0}^{p}}}{2} \end{aligned}$$

Notice that the basic reproduction number $R_0$ consists of two parts, representing the two modes of transmission of the coronavirus.

### Results

**Fig. 2:**
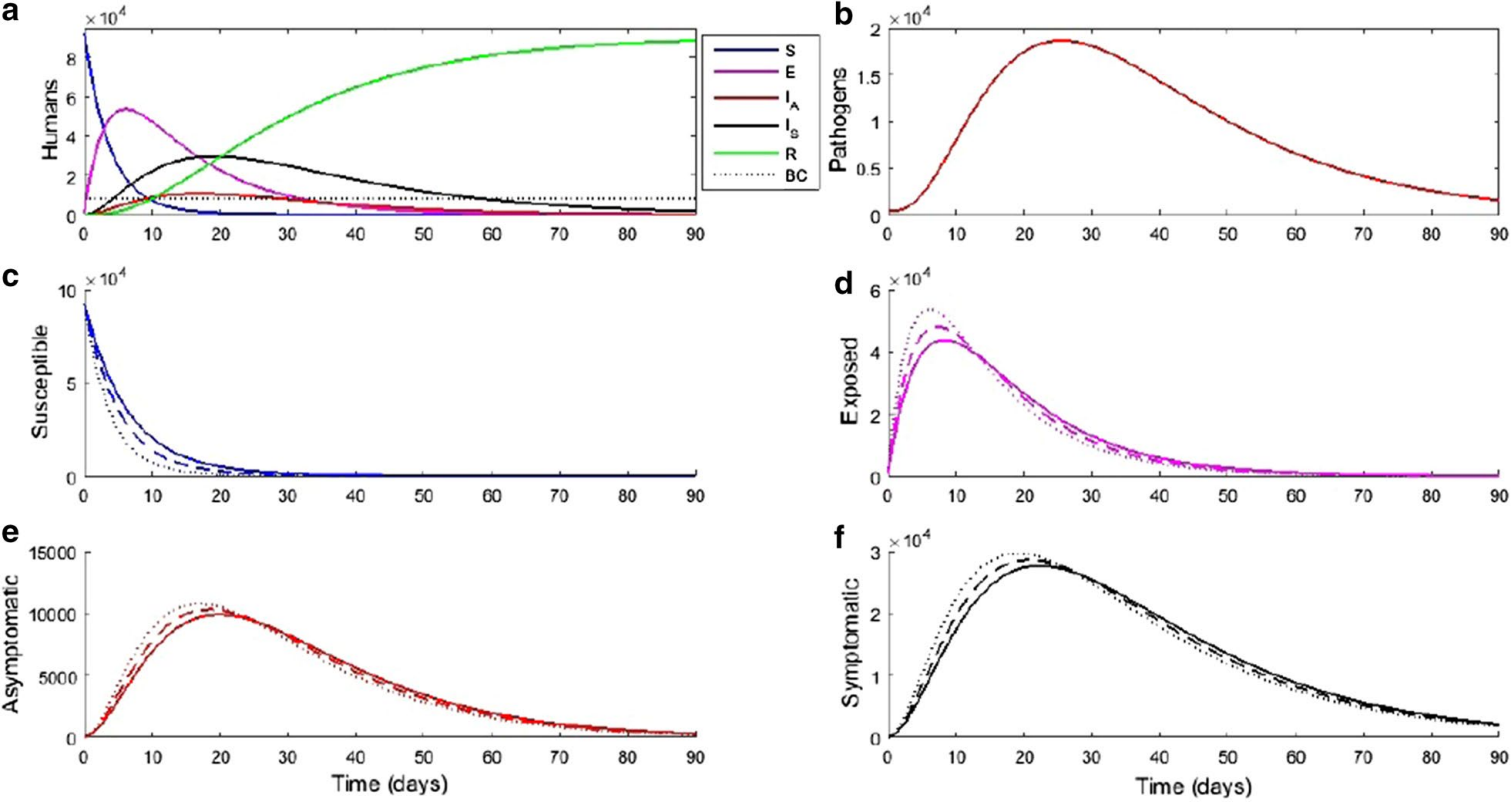
The simulated humans and pathogens populations are shown in (**a**, **b**) respectively. Effects of the constants $\alpha _1$ and $\alpha _2$ which determines the rate of new infections, are shown in (**c**–**f**): $\alpha _1=0.1, \alpha _2=0.1$, is depicted by the continuous line, $\alpha _1=0.05, \alpha _2=0.1$ is depicted by the dashed line, and $\alpha _1=0.1, \alpha _2=0.05$ is depicted by the dotted line

In this section, we approximate solutions to the model equations (1) using fourth and fifth order Runge–Kutta methods which are implemented via the *ode45* function in MATLAB. The initial values used are $S(0)=93000,E(0)=1000,I_A(0)=50,I_A(0)=50,I_S(0)=50,R(0)=0,P(0)=500$. Figure 2a depicts the change in the populations as time increases from 0 to 90 days. During the first 10 days, the number of susceptible humans declines rapidly as the number of exposed individuals increases rapidly due to contact with infected individuals ($I_A$ and $I_S$) and also the virus in the environment (*P*). After the latency period, and without mitigating the epidemic, the number of infected individuals surges, surpassing the hospital bed capacity, set here as 8000. The infected individuals who exhibit mild or no symptoms $I_A$ are considered to be 30% of the total infected population. The model parameters used in this simulation study are shown in Table 1.

Since the symptomatic individuals $I_S$ are assumed to be more infectious than the asymptomatic $I_A$, the transmission of COVID-19 through contacts in households, workplaces, schools, from foodstuffs, or during commute rises. This leads to a surge of the virus in environments such as workplace, school, foodstuffs, and public transport, see Fig. 2b, and consequently more cases of the coronavirus are confirmed, see Fig. 2a between 10 and 35 days. In this model we take the constants $\alpha _1$ and $\alpha _2$ to be reciprocal of the frequency with which individuals acquires the COVID-19 from the environment and from infected individuals, respectively. In Fig. 2 the model shows that when $\alpha _1 =0.05$ i.e. there is a high risk of getting infected by a contaminated environment, as compared to an infected individual, the number of exposed, asymptomatic and symptomatic individuals increases. However, when $\alpha _2 =0.05$ i.e. higher chances of getting infected by an individual, as compared to a contaminated environment. Moreover, in Fig. 2c the number of susceptible vanishes by the 23rd day for $\alpha _2=0.05$ since many people were infected quite rapidly, see Fig. 2d–f for duration $0-20$ days. Therefore, with very low new infections the number of infected individuals subsequently reduces from the 25th day onward, where the number of infected individuals is seen to be lower for $\alpha _2=0.05$, as compared to when $\alpha _1=0.05$ and $\alpha _2=0.1$.

### Discussion

The model shows that control measures such as social distancing, wearing of masks in public, frequent hand washing and limiting non-essential travel needed to avoid a large COVID-19 epidemic. There is a growing concern that this disease could continue to ravage the human population globally since many aspects of the COVID-19 are yet to be discovered, which also poses a challenge to the long-term mathematical modeling of the disease.

## Limitations

This model was designed to look at transmission dynamics so does not describe disease severity and death. Given we made assumptions of the parameters at onset of the pandemic, there is a possibility that the model may overestimate the pandemic at later period of time.

## Data Availability

The data used to support findings of this study is available from the corresponding author upon request.

## Abbreviations

COVID-19: Coronavirus disease 2019
MERS-CoV: Middle East respiratory syndrome coronavirus
SARS: Severe acute respiratory syndrome
SARS-CoV-2: Severe acute respiratory syndrome coronavirus 2
SEIR: Susceptible exposed infectious recovered
WHO: World Health Organization
